# MicroRNAs in the Pathogenesis of Hepatocellular Carcinoma: A Review

**DOI:** 10.3390/cancers13030514

**Published:** 2021-01-29

**Authors:** Asahiro Morishita, Kyoko Oura, Tomoko Tadokoro, Koji Fujita, Joji Tani, Tsutomu Masaki

**Affiliations:** Department of Gastroenterology and Neurology, Faculty of Medicine, Kagawa University, Kagawa 761-0793, Japan; kyoko_oura@med.kagawa-u.ac.jp (K.O.); t-nishioka@med.kagawa-u.ac.jp (T.T.); 92m7v9@med.kagawa-u.ac.jp (K.F.); georget@med.kagawa-u.ac.jp (J.T.); tmasaki@med.kagawa-u.ac.jp (T.M.)

**Keywords:** microRNA, hepatocellular carcinoma, pathogenesis, biomarker, therapeutic target

## Abstract

Hepatocellular carcinoma (HCC) is the seventh most frequent cancer and the fourth leading cause of cancer mortality worldwide. Despite substantial advances in therapeutic strategies, the prognosis of late-stage HCC remains dismal because of the high recurrence rate. A better understanding of the etiology of HCC is therefore necessary to improve outcomes. MicroRNAs (miRNAs) are small, endogenous, noncoding, single-stranded RNAs that modulate the expression of their target genes at the posttranscriptional and translational levels. Aberrant expression of miRNAs has frequently been detected in cancer-associated genomic regions or fragile sites in various human cancers and has been observed in both HCC cells and tissues. The precise patterns of aberrant miRNA expression differ depending on disease etiology, including various causes of hepatocarcinogenesis, such as viral hepatitis, alcoholic liver disease, or nonalcoholic steatohepatitis. However, little is known about the underlying mechanisms and the association of miRNAs with the pathogenesis of HCC of various etiologies. In the present review, we summarize the key mechanisms of miRNAs in the pathogenesis of HCC and emphasize their potential utility as valuable diagnostic and prognostic biomarkers, as well as innovative therapeutic targets, in HCC diagnosis and treatment.

## Simple Summary

Hepatocellular carcinoma (HCC) is one of the most frequently occurring cancers, and the prognosis for late-stage HCC remains poor. A better understanding of the pathogenesis of HCC is expected to improve outcomes. MicroRNAs (miRNAs) are small, noncoding, single-stranded RNAs that regulate the expression of various target genes, including those in cancer-associated genomic regions or fragile sites in various human cancers. We summarize the central roles of miRNAs in the pathogenesis of HCC and discuss their potential utility as valuable biomarkers and new therapeutic agents for HCC.

## 1. Introduction

Liver cancer is the sixth most frequent malignancy and the fourth leading cause of cancer-related deaths worldwide [1]. Hepatocellular carcinoma (HCC) accounts for approximately 80% of all liver cancers and is a main cause of cancer mortality [2]. The risk factors of hepatocarcinogenesis include various liver diseases, such as infections caused by hepatitis B virus (HBV) and hepatitis C virus (HCV), alcoholic liver disease (ALD), nonalcoholic steatohepatitis (NASH), and nonalcoholic fatty liver disease (NAFLD) [3,4,5]. Despite recent great progress in HCC therapy, the 5-year survival rate for advanced-stage HCC remains poor owing to its late diagnosis, resistance to anticancer therapy, and high frequency of recurrence [6,7,8]. Therefore, elucidating the detailed underlying mechanisms and pathogenesis of HCC is important for the development of new diagnostic and prognostic biomarkers and therapeutic drugs.

MicroRNAs (miRNAs, miRs) are small, endogenous, interfering, noncoding RNAs of 21–30 nucleotides in length. More than 2600 miRNAs have been predicted to be encoded by the human genome, with the ability to modulate more than 15,000 genes [9]. Each miRNA negatively regulates target genes by binding to the 3′ untranslated region (UTR) of mRNAs. These complexes are involved in RNA-mediated interference, and in vertebrates, mRNA transcripts are usually not cleaved by a miRNA-associated RNA-induced silencing complex (RISC) but rather undergo translational repression and degradation via deadenylation [10]. The miRNA-associated RISC can suppress gene expression [11,12]. Remarkably, a single miRNA can modulate more than 200 mRNAs [13,14].

**Figure 1:**
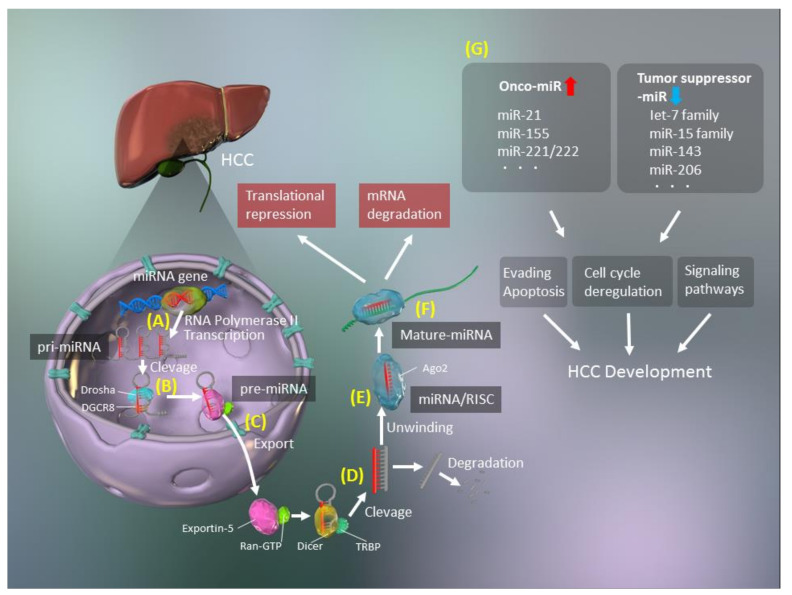
Schematic of microRNA (miRNA, miR) biogenesis. (**A**) Synthesis of primary miRNA (pri-miRNA) transcripts from genomic DNA by RNA polymerase II. (**B**) The pri-miRNA is cleaved by Drosha/DiGeorge syndrome critical region 8 (DGCR8) and processed to a precursor miRNA (pre-miRNA). (**C**) The pre-miRNA forms a complex with exportin-5 and Ran-GTP and is exported to the cytoplasm. (**D**) The exported hairpin pre-miRNA is cleaved by Dicer/transactivation response element RNA-binding 70 protein (TRBP). (**E**) The double-stranded miRNA is unwound and forms an RNA-induced silencing complex (RISC) with Argonaute 2 (AGO2). (**F**) The miRNA is separated into a mature, single-stranded miRNA. (**G**) Upregulation of oncogenic miRs (oncomiRs) and downregulation of tumor suppressor miRs promote hepatocellular carcinoma (HCC) development.

The biogenesis of miRNAs includes several steps, such as transcription, cleavage, export, further cleavage, strand selection, and interaction with mRNAs [15] (Figure 1). Generally, RNA polymerase II transcribes primary miRNA (pri-miRNAs) transcripts in the canonical pathway or mirtron pathway [16]. The 5′ ends of pri-miRNAs are capped, and the 3′ ends are polyadenylated [17]. Pri-miRNAs are then cleaved to 70–100-nucleotide hairpin-structured precursors (pre-miRNAs) by a nuclear RNase III enzyme named Drosha, which is a double-stranded RNA-binding domain protein involving DiGeorge syndrome critical region 8 (DGCR8)/Pasha as a cofactor [18]. Next, pre-miRNAs are exported from the nucleus to the cytoplasm, after binding with exportin-5 and Ran-GTP [19] and are cleaved by Dicer [20], with transactivation response element RNA-binding 70 protein (TRBP) serving as a cofactor for Dicer [20]. Finally, double-stranded pre-miRNAs undergo rapid unwinding, when loaded onto Argonaute (AGO), and only one strand, which serves as a guide to target mRNAs, remains bound [21]. These RISCs with the AGO protein modulate mRNA degradation and translational inhibition [21].

However, the biogenesis of miRNAs is more diverse and may involve noncanonical pathways that bypass Drosha/DGCR8 processing. There are several noncanonical types of miRNAs, including mirtrons, small nucleolar RNAs (snoRNAs), transfer RNAs (tRNAs), and short hairpin RNAs (shRNAs) [22]. Mirtrons are pre-miRNAs that are generated without Drosha by pre-mRNA splicing and intron debranching [23,24]. SnoRNAs [25] and tRNAs [26,27] have different internal hairpin structures, and their processing involves the cleavage activity of Dicer, without Drosha/DGCR8. Transcripts of shRNA, which are derived from unannotated and intergenic regions, can function as Dicer substrates during transcription [28,29].

Although the important roles of miRNAs in the modulation of mRNA expression are well established, their precise functions remain elusive. Interestingly, miRNAs modulate the expression of approximately 30% of all human genes, many of which are tumor-associated or are in regions of instability in the genome [30,31]. There is clear evidence of key roles for miRNAs in human carcinogenesis [13,32,33,34,35], with two types of miRNAs identified, namely, oncogenic miRNAs (oncomiRs) and tumor suppressor miRNAs (Figure 1). OncomiRs induce carcinogenesis by inhibiting the expression of tumor suppressors, while tumor suppressor miRNAs inhibit oncogene expression in normal cells and are lacking in cancer cells. Two miRNAs, miR-15 and miR-16, were first reported to be altered in cancer and are associated with a frequently targeted chromosomal deletion of *BCL2*, which encodes an anti-apoptotic factor [36].

Several reports have demonstrated relationships between miRNAs and HCC [35,37,38,39,40] and identified potential miRNA biomarkers and therapeutic targets for HCC diagnosis and treatment. This review provides a summary of (1) miRNA functions in the liver, (2) associations of miRNAs with the pathogenesis of HCC of various etiologies, and (3) the mechanisms underlying the miRNA effects. Furthermore, we emphasize potential utility of various miRNAs as HCC biomarkers and target molecules for therapeutics.

## 2. Roles of miRNAs in the Liver

### 2.1. Lipid Metabolism

The liver plays a critical role in lipid metabolism. Dysfunctions in lipid metabolism induce excessive accumulation of hepatic triglycerides and fatty acids, resulting in various liver diseases, such as NAFLD and NASH. Several miRNAs have pivotal functions in the maintenance of cholesterol and fatty acid metabolism [41], and others, such as miR-33, miR-103, miR-104, and miR-307, act as modulators of lipid and cholesterol levels [42].

Various serum miRNAs, including miR-122, miR-21, miR-34a, and miR-451, are enhanced in patients with NAFLD [43]. A representative liver-specific miRNA, miR-122, which is highly upregulated in the liver, is involved in hepatic cholesterol and lipid metabolism in this disease [44]. Suppression of miR-122 can diminish the plasma cholesterol levels, reduce hepatic fatty acid and cholesterol synthesis, and increase the oxidation of hepatic fatty acids [45]. In addition, miR-122 is associated with hepatic lipogenesis-related enzymes, including fatty acid synthase (FASN) and acetyl-CoA carboxylase (ACC1). The inhibition of miR-122 also decreases hepatic lipogenesis in obese mice [44].

Several reports have demonstrated that miR-34a is associated with hepatic steatosis [46,47]. This miRNA inhibits hepatic silent information regulator 1 (sirtuin 1, SIRT1), peroxisome proliferator-activated receptor-α (PPARα), and liver X receptor (LXR) [46,47,48]. Reduced SIRT1 levels in the liver of patients with NAFLD can be recovered by the inhibition of miR-34a, which results in the improvement of hepatic steatosis via PPARα and the activation of AMP-activated protein kinase (AMPK) [47].

### 2.2. Glucose Metabolism

miR-375, a novel islet-specific miRNA, can inhibit glucose-induced insulin secretion, whereas inhibition of miR-375 can increase insulin secretion. This miR-375-associated insulin modulation is independent of altered glucose metabolism and is related to insulin exocytosis. Myotrophin (MTPN), which is a target of miR-375, is also involved in glucose metabolism. The inhibition of MTPN enhances glucose-induced insulin secretion and insulin exocytosis, indicating that miR-375 might be a new therapeutic target for diabetes mellitus and NAFLD [49]. Expression of miR-23a is increased in a NASH-related HCC murine model, and overexpression of miR-23a via the interleukin-6 (IL-6)/STAT3 signaling pathway decreases glucose production through the inhibition of *PGC1*α and *G6PC* expression [50]. In addition, miR-143, which is involved in insulin resistance, controls the ORP8-dependent regulatory pathway of AKT, and miR-143 overexpression reduces insulin-stimulated AKT activation [51]. Moreover, miR-206 decreases lipid and glucose levels in hepatocytes by modulating lipogenesis and insulin signaling [52], which suggests that miR-206 might be a diagnostic biomarker and therapeutic target for NAFLD and hyperglycemia.

### 2.3. Hepatic Inflammation

Hepatic inflammation, which involves inflammatory cytokine production and endoplasmic reticulum stress, results from an abnormal immune response; the latter is mediated by several factors, such as viral and bacterial infections, metabolic disorders, alcohol abuse, drug allergies, and toxic reagents [53]. Various miRNAs, such as miR-122 and miR-132, play pivotal roles in the innate and adaptive immunity involved in hepatic inflammation. miR-122 is the most representative miRNA in the liver, and its loss results in inflammation, fibrosis, and HCC, indicating that miR-122 modulates anti-inflammatory effects [54,55]. Furthermore, miR-122 may inhibit hepatic infiltration of inflammatory cells and the secretion of various cytokines, including IL-6 and tumor necrosis factor-α (TNFα), by these cells [54,55]. miR-132, which inhibits the expression of SIRT1, is a mediator of inflammation in chronic liver diseases. The overexpression of miR-132 induces the translocation of nuclear factor-κB (NF-κB) into the nucleus, acetylation of p65, and production of IL-8 and MCP-1. The loss of miR-132, which is mediated by serum deprivation, diminishes the acetylation level of p65 and partially downregulates the expression of IL-8 and MCP-1. Therefore, the inhibition of miR-132 has anti-inflammatory effects in the liver.

### 2.4. Hepatic Fibrosis

Fibrosis is developed in the liver as a result of continuous and severe hepatocyte damage, resulting in an inflammatory cytokine storm. Several miRNAs can synergistically modulate inflammatory signaling pathways. Furthermore, several miRNAs are associated with the activation of hepatic stellate cells (HSCs) and progression of hepatic fibrosis via the regulation of related signaling pathways.

Members of the miR-29 family induce apoptosis via the phosphatidylinositol 3-kinase (PI3K)/AKT signaling pathway and modulate the accumulation of the extracellular matrix (ECM) [56,57,58]. Stimulation of HSCs by transforming growth factor-beta (TGF-β) promotes myofibroblastic transition and ECM induction, resulting in liver fibrogenesis. TGF-β1 mediates the downregulation of miR-29 in HSCs [57]. In addition, miR-29 overexpression in mouse HSCs leads to the reduction of collagen-1α1 and collagen-4α1 levels [57,59,60] via modulation of various ECM genes. Similar to other hepatic fibrosis-related miRNAs, members of the miR-34 family promote hepatic fibrosis by activating HSCs, whereas those of the miR-378 family inhibit the development of fibrosis in a GLIS-dependent manner. The miR-15 family is associated with the induction of cell proliferation and apoptosis, and its members regulate the TGF-β signaling pathway by inhibiting TGFβR1, SMAD3, SMAD7, p38, and endoglin in cardiac fibrosis [61]. The miR-199 and miR-200 families are involved in the secretion of profibrotic cytokines and are responsible for ECM deposition [62,63]. These miRNA families act as modulators of hepatic fibrosis by targeting genes involved in fibrosis-related signaling pathways and activating HSCs.

## 3. Roles of miRNAs in Various Liver Diseases Leading to HCC

### 3.1. HBV Infection

HBV infection is one of the most prevalent risk factors of HCC development [64]. Thus, HBV-related HCC is a serious health concern worldwide [64]. Recently, many reports have demonstrated that miRNAs play pivotal roles in each stage of HBV-related HCC development [65,66,67,68]. HBV dysregulates miRNAs that modulate the expression of the host/HBV genes during HCC pathogenesis. In fact, this type of HCC is characterized by a range of immune response failures due to the dysregulation of miRNAs [69].

The HBV X protein (HBx) inhibits miR-34 expression via p53 stimulation in hepatocytes, which results in the upregulation of a macrophage-derived chemokine (CCL22), stimulation of regulatory T cells (Tregs), and the suppression of effector T cells, thereby increasing HBV genome transcription [70,71]. HBx-induced upregulation of miR-155 leads to a reduction in the suppressor of cytokine signaling-1 (SOCS1) expression, increasing JAK/STAT signaling and suppressing HBV infection mediated by the induction of interferon (IFN) signaling [72]. In addition, HBx-induced miR-155 blocks CCAAT/enhancer-binding protein (C/EBP), which activates the HBV enhancer (Enh) 11/core promoter and then inhibits HBV replication [73].

Liver-specific miR-122 is upregulated in the serum after HBV infection and is regarded as one of the key modulators of HBV replication [74,75,76]. However, Wang et al. [77] demonstrated that HBx suppressed miR-122 expression and increased the levels of HBV transcripts by blocking p53 binding to the HBV Enh1/core promoter. Another report has revealed that miR-122 blocks HBV pregenome RNA, which encodes the hepatitis B core antigen and viral polymerase and inhibits HBV replication via dysregulation of heme oxygenase-1 (HO-1), which suppresses refill of HBV covalently closed circular DNA [78]. In addition, upregulation of several miRNAs, including miR-184, miR-185, miR-196a, miR-199a-3p, miR-210, and miR-217, directly affects HBV transcription [79]. Furthermore, it has recently been demonstrated that the HBV virion produces its own HBV-miR-2 and HBV-miR-3 [80]. HBV-miR-3 inhibits mRNA expression of the hepatitis B core (HBc) protein, which is involved in HBV self-regulation [80], to prolong survival by escaping the host immune system [81,82]. miR-372 also modulates HBV expression, which depends on target pathways. HBx-upregulated miR-372 [83] targets the cyclic AMP response element-binding protein (CREB) by binding to HBV Enh1/core promoter (ENI-Cp), thereby suppressing HBV transcription. miR-372 targets nuclear factor 1 B-type protein (NFIB), which modulates HBV Enh1/ENI-Cp, thus increasing HBV transcript levels [84]. Various miRNAs are thus related to the modulation of HBV transcript levels and the host immune response to the virus.

### 3.2. HCV Infection

HCV contributes to serious liver problems, such as liver cancer, including HCC [85]. IFN-free, direct-acting antiviral therapy improves HCV treatment, with sustained virologic response rates greater than 95%, without many side effects [86]. However, the high mutation rate of HCV may result in the resistance to direct-acting antivirals, and patients with mutated HCV have low sustained virologic response rates [87]. Therefore, further studies of the molecular mechanism underlying HCV infections are needed to improve HCV therapy.

Dysregulation of miRNAs due to HCV infection occurs via multiple pathways, such as the immune response, lipid metabolism, and cell-cycle pathways [88]. Several target genes, such as *PPARG* and fibronectin 1 (*FN1*), were found to be downregulated, while other target genes, including stearoyl-CoA desaturase (*SCD*) and *CREB1*, were found to be upregulated by at least 11 miRNAs, namely, miR-130a/b, miR-200, miR-34a, miR-23b, miR-24, miR-146a, miR-381, miR-25, miR-200a, and miR-371-5p, after HCV infection [89]. In addition, miR-122 modulates the host immune response by regulating the expression of target genes and directly targeting the HCV genome [90,91,92]. In particular, miR-122 stabilizes the 5′ and 3′ UTRs of the HCV genome, and inhibition of this miRNA dramatically reduces the replication of HCV RNA [93,94]. By contrast, miR-141, miR-192, miR-215, and miR-491 increase HCV replication. Thus, miR-141 enhances HCV replication by reducing the tumor suppressor deleted in liver cancer 1 (DLC-1) [95], and miR-491 enables HCV entry via the PI3K/AKT signaling pathway [96]. However, miR-196, miR-29, let-7b, miR-130a, and miR-27a induce anti-HCV activity [97,98,99,100,101]. It has also been found that miR-199a suppresses HCV replication by blocking domain II of the internal ribosomal entry site [97]. In addition, IFN-β enhances the expression of several miRNAs with anti-HCV activity, such as miR-196, miR-431, and miR-448. Among these, miR-196 and miR-448 may directly target the HCV RNA genome [91]. These findings suggest that several miRNAs are strongly involved in the modulation of HCV infection and replication.

### 3.3. ALD

ALD is a complex disease caused by prolonged and heavy alcohol consumption, along with predisposing genetic factors. ALD can cause liver dysfunction, including steatohepatitis, fibrosis, cirrhosis, and eventually HCC [102]. There is tremendous evidence of the effects of miRNA dysregulation on the pathogenesis and development of ALD, such as liver damage, lipid metabolism dysfunction, inflammation, oxidative stress, apoptosis, and fibrosis [102]. Inflammation-related miRNAs, such as miR-132, miR-155, miR-146, and miR-21, influence alcohol/lipopolysaccharide (LPS)/TLR4 pathways. TLR4 transmits proinflammatory stimuli via a mitogen-activated protein kinases (MAPKs) or TIR domain-containing adaptor-inducing IFN-β (TRIF) [103]. The expression of miR-212 is increased by alcohol in intestinal cells, and LPS is also induced by alcohol in the liver via the suppression of ZO-1 [104,105]. Alcohol-induced oxidative stress downregulates miR-199a in liver sinusoidal endothelial cells by upregulating ET1 and hypoxia-inducible factor 1α (HIF1α), which are related to steatohepatitis and fibrosis in the liver [106]. The downregulation of miR-223 increases neutrophil infiltration in the liver and induces liver injury by inhibiting the IL-6/p47^phox^/reactive oxygen species axis [107]. The enhanced expression of miR-217 exacerbates alcoholic fatty changes by damaging SIRT1 and lipin-1 [108]. Moreover, upregulation of miR-34a in ALD results in the development of liver fibrosis via caspase-2, SIRT1, and matrix metallopeptidase (MMP) 1 and MMP2 [108]. In addition, miR-122 modulates hepatic lipid metabolism and inflammation [109]. The miRNA let-7 is diminished in response to alcohol and loss of let-7 induces a mesenchymal phenotype in HSCs to enhance liver injury by inhibiting LIN28B. Finally, an imbalance between let-7 and LIN28/28B causes oncogenesis [110].

### 3.4. NAFLD and NASH

NAFLD is defined as steatosis in at least 5% of hepatocytes [111], without other liver diseases, including viral hepatitis and autoimmune, alcohol-related, and genetic liver diseases. Recently, NAFLD has become one of the most frequent liver diseases worldwide [112]. Various miRNAs have been identified to be involved in NASH development. For example, the levels of miR-122, which is a representative hepatic miRNA, are 7.2 times higher in patients with NASH than in healthy subjects and 3.1 times higher than in patients with simple steatosis [113]. Liver-specific miR-122-knockout mice rapidly develop NASH and exhibit enhanced lipogenesis, changes in lipid secretion, IL-6 and TNF-α production, and upregulation of chemokine (C–C motif) ligand 2 (CCL2). Decreased miR-122 expression enhances fibrogenesis by inducing HIF1α and MAPK1, which can also facilitate HCC development [114]. In addition, miR-192 is involved in the development of TGF-β1-promoted fibrosis, which activates SMAD signaling [115]. However, members of a miRNA superfamily that includes miR-16, miR-497, miR-195, miR-322, and miR-15, particularly miR-15 and miR-16, regulate hepatic fibrosis and hepatocarcinogenesis [61]. The development and progression of NASH increases the risk of HCC via miRNAs. A recent report has demonstrated that miRNAs are pivotal for the activation of HSCs during NASH development [116]. Free cholesterol is accumulated because of increases in both SREBP2 and miR-33a signaling via the inhibition of PPARγ signaling, along with HSC activation and disruption of the SREBP2-induced cholesterol feedback system [116]. Upregulation of miR-21, which downregulates the expression of the tumor suppressor phosphatase and tensin homolog (PTEN), is mediated by unsaturated fatty acids in hepatocytes [117]. Furthermore, the expression of miR-155, which inhibits another tumor suppressor gene, C/EBPβ, is enhanced in mice fed a choline-deficient, amino acid-defined diet [73,118].

## 4. Dysregulation of miRNAs and HCC Development

### 4.1. miRNAs Involved in Carcinogenesis

Dysregulation of miRNAs is observed in various cancer types [119,120,121,122,123,124]. Although several tumor suppressor miRNAs, which target oncogenes, are downregulated, other miRNAs (oncomiRs), which target tumor suppressor genes, are upregulated in HCC. Genomic regions encoding miRNAs associated with dysfunction can protect against genetic mutations. Carcinogenesis-related transcription factors, such as MYC, suppress some miRNAs, whereas epigenetic regulation of other miRNAs occurs via DNA methylation and histone modifications [125]. In addition, the loss of miRNA processing machinery genes, such as *DROSHA*, *DGCR8*, *DICER1*, *TRBP*, and *AGO2*, reduces mature miRNA synthesis and results in hepatocarcinogenesis and HCC development [125]. Various HCC-associated miRNAs, including miR-21, miR-221, and miR-222, are increased in HCC [35,38,39], whereas others, such as miR-122a, miR-145, miR-199a, and miR-223, are decreased in this disease [37,68]. Several HCC-specific miRNAs have recently been classified (Table 1). In our previous study, we demonstrated that miRNA profiles differed between hepatocytes and HCC cells [126]. This finding suggests that the study of miRNA profiles during HCC development and progression can provide valuable biomarkers and therapeutic targets.

### 4.2. miRNAs Associated with HCC Development

miRNAs have pivotal functions in cancer development [279], and their profiles differ between normal tissues and various cancers. Many reports have demonstrated that the ectopic expression of miRNAs has oncogenic or tumor-suppressive effects on HCC [280,281,282,283,284,285,286]. Therefore, these miRNAs might be targets for HCC therapy.

#### 4.2.1. Upregulated miRNAs Involved in HCC Development (Oncogenic miRNAs)

miR-21, an oncogenic miRNA, is a potential diagnostic biomarker for HCC and is frequently overexpressed in various cancers [15,287]. In addition to HCC, it is upregulated in several liver diseases, such as viral hepatitis, NAFLD, ALD, and liver fibrosis [288]. miR-21 promotes fibrosis by activating HSCs, which results in hepatocarcinogenesis [289,290,291,292]. In patients with HCC, the expression levels of miR-21 are significantly upregulated in both serum and tissues [288,293] and are correlated with tumor development [294]. Additionally, enhanced miR-21 expression promotes cancer cell migration and invasion by inhibiting Krüppel-like factor 5 (KLF5) in vitro [293]. Chen et al. [295] demonstrated that secreted exosomal miR-21 activated the pyruvate dehydrogenase kinase 1 (PDK1)/AKT pathway in HSCs located near HCC (cancer-associated fibroblasts) by directly targeting PTEN and promoted cancer progression by inducing the secretion of angiogenic molecules, such as vascular endothelial growth factor (VEGF), basic fibroblast growth factor (bFGF), MMP2, MMP9, and TGF-β by HCC cells.

miR-155 is an inflammation-related miRNA in various liver diseases, including hepatitis B and NASH [296,297,298], which enhances liver carcinogenesis [299]. miR-155 plays important roles as an oncomiR by inhibiting various tumor suppressors, such as SOCS1 [300], tumor protein p53-inducible nuclear protein 1 (TP53INP1) [301], and mutL homolog 1 (MLH1) [302]. In addition, miR-155 overexpression enhances cell proliferation via activation of the Wnt/β-catenin [303], AKT [304], and JAK2/STAT3 [305] pathways. However, Chen et al. [306] demonstrated that in human cancer, miR-155 could act as a tumor suppressor by modulating claudin-1 (CLDN1) and SMAD2 and as an oncomiR under different circumstances.

miR-221 and miR-222 are the most highly deregulated miRNAs in HCC tissues [254]. miR-221 targets key tumor suppressors, including CDKN1B/p27 [307,308,309], CDKN1C/p57 [310,311], and DNA damage-inducible transcript 4 (DDIT4) [254]. Callegari et al. [251] developed transgenic models overexpressing miR-221/222 in the liver and confirmed that dysregulation of these miRNAs led to hepatocarcinogenesis. These studies suggest that miR-221 is a key oncogenic miRNA in HCC.

#### 4.2.2. Downregulated miRNAs Involved in HCC Development (Tumor Suppressors)

Tumor suppressor miRNAs, including the let-7 family, target the RAS family [287,312,313]. Members of the let-7 family exert antifibrotic effects at the transcriptional level via PPARγ. These miRNAs regulate various target genes associated with liver fibrosis, thereby diminishing tumorigenesis [314,315]. Remarkably, expression of the members of the let-7 family is diminished in HCV-associated HCC [135]. Among let-7 family members, let-7g levels are notably lower in metastatic HCC than in HCC without metastasis, while higher expression of let-7g in HCC tissues predicts a better prognosis in patients with HCC [135].

Members of the miR-15 family have a common seed sequence and inhibit specific mRNAs. In HCC tissues, members of this family are diminished as tumor suppressors and modulate inflammation by inhibiting IκB kinase (IKKα) and TGF-β-activated kinase 1 binding protein 3 (TAB3) [316]. miR-195, which is in the miR-15 family, suppresses angiogenesis by targeting VEGF, Vav guanine nucleotide exchange factor 2 (VAV2), and cell division cycle 42 (CDC42) in HCC cell lines [317]. A recent report has also demonstrated that miR-497, which also belongs to the miR-15 family, modulates the protein kinase B (PKB) pathway by targeting RICTOR in the HCC cell lines and inhibits cell growth, invasion, and metastasis via the RICTOR/AKT signaling pathway [217].

miR-29 acts as a tumor suppressor in several cancers and regulates oncogenic processes, including epigenetic modifications, proliferation, apoptosis, fibrosis, and angiogenesis [318]. Overexpression of miR-29 induces apoptosis and inhibits tumorigenesis by directly inhibiting anti-apoptotic proteins, including BCL2 and MCL1, based on an in vivo study of HCC [319]. Loss of miR-101, which targets MCL1, induces apoptosis and inhibits tumorigenesis [156]. Furthermore, miR-101 targets Rho-associated protein kinase (ROCK), which is a downstream effector of RhoA GTPase and modulates actomyosin bundles and focal adhesions involved in the inhibition of epithelial–mesenchymal transition (EMT). The overexpression of ROCK2, which is frequently detected in HCC tissues, induces cancer cell motility and invasiveness [320].

miR-122, which is a representative miRNA in the liver, plays a critical role in lipid metabolism and HCV replication, as well as in hepatocarcinogenesis. Thus, miR-122 directly inhibits cyclin G1, IGF-1R, ADAM10, and pyruvate kinase M2 (PKM2) as a tumor suppressor in HCC [321]. Interestingly, in miR-122-knockout mice, the loss of miR-122 causes steatohepatitis and carcinogenesis in the liver [54]. Furthermore, restoration of miR-122 prevents hepatocarcinogenesis in a mouse model [321].

#### 4.2.3. miRNAs and the Tumor Microenvironment (Angiogenesis and Immune Modulation)

HCC is a hypervascular tumor, and angiogenesis is one of the most important factors during HCC development, including proliferation, invasion, and metastasis of the tumor. VEGFA is a critical mitogen for endothelial cells and plays an important role in tumor vessel formation [322]. Several studies have demonstrated that the expression of VEGFA is upregulated in HCC and is associated with oncogenesis [323,324]. Some miRNAs, such as miR-15b, miR-125b, miR-423-3p, miR-424, miR-494, miR-497, miR-612, miR-637, and miR-1225b, are involved in angiogenesis, including modulation of VEGF expression [325].

Recent reports have demonstrated that some specific miRNAs could play important roles in the tumor microenvironment during HCC development [71]. TGF-β inhibits miR-34a expression, resulting in chemokine CCL22 upregulation, which increases the recruitment of Tregs to facilitate immune escape [71]. In addition, Tregs exhibit altered expression of several specific miRNAs, including miR-182-5p, miR-214-3p, miR-129-5p, and miR-30b-5p, and are influenced by FOXP3 in HCC [326,327]. Li et al. [328] also demonstrated that miR-125b inhibited Treg accumulation and functions induced by *Ganoderma lucidum* polysaccharide extract, resulting in the suppression of HCC growth. These findings suggest that the expression of specific miRNAs is associated with tumor growth, not only from cancer cells but also from stromal cells, including endothelial and immune cells, during HCC development.

### 4.3. miRNAs as Biomarkers for HCC

The prognosis of HCC remains poor, as many patients are diagnosed at an advanced stage. Tumor markers, such as α-fetoprotein (AFP) and protein induced by vitamin K absence or antagonists-II (PIVKA-II), are widely used for HCC screening. However, the diagnosis of HCC using these tumor markers has yielded unsatisfactory results [329]. Therefore, new effective biomarkers are required, especially those involved in early HCC.

Many studies have demonstrated the diagnostic and predictive value of several miRNAs in HCC (Table 2). Circulating miRNAs are stably detected in the blood [330] and are therefore one of the most valuable biomarkers for HCC. Basically, miRNAs secreted from cancer cells are contained in exosomes or apoptotic bodies or are bound to serum proteins or lipids [331]. Among miRNAs that are altered in HCC, the downregulated miRNAs, including let-7g [135], miR-22 [332,333,334], miR-26 [334], miR-29 [319], miR-99a [154], miR-122 [335], miR-124 [336], miR-139 [337], miR-145 [179], and miR-199b [193], are associated with cell growth, apoptosis, carcinogenesis, angiogenesis, migration [338], invasion, metastasis, and EMT (Table 2). Upregulated miRNAs, such as miR-10b [227], miR-17-5p [339], miR-21 [340], miR-135a [236], miR-155 [341], miR-182 [243], miR-221 [340], and miR-222 [170,340], are associated with metastasis, angiogenesis, and a poor prognosis (Table 2). Additionally, miR-500 is upregulated in the serum of patients with HCC and is downregulated after surgical resection of the tumor [342]. Moreover, miR-25, miR-375, and let-7f can also be used to distinguish patients with HCC from normal controls [343]. These findings indicate that miRNAs can be promising new biomarkers for HCC.

### 4.4. miRNAs as Therapeutic Targets for HCC

Various miRNAs have been demonstrated to modulate multiple targets in HCC-associated signaling pathways, and miRNA-targeted cancer treatment is an attractive approach for the prevention of HCC development. Recently, reduced miRNA expression in HCC has been found to be restored with replacement therapy, leading to the inhibition of transcription of critical mRNAs that suppress cell proliferation or induce apoptosis in HCC cell lines [348]. By contrast, enhanced expression of miRNAs can be inactivated by antagonists in HCC cells [349]. However, the administration of miRNAs has not resulted in sufficient gene knockdown, since cell membrane permeability is poor, and the nucleus is degraded. To overcome these obstacles, miRNA oligomers, including anti-miRNA oligonucleotides, locked nucleic acid antisense oligonucleotides, miRNA sponges, miRNA masks, nanoparticles, antagomirs, and multiple-target anti-miRNA antisense oligodeoxyribonucleotides, have recently been used to modulate miRNAs [350,351]. Targeting of candidate miRNAs has been developed as a new approach for HCC therapy.

Adeno-associated virus (AAV) vectors can be utilized for the delivery of miRNA inhibitors or miRNA precursors. Notably, miR-26a, which is expressed at low levels in HCC tissues and at high levels in the normal liver, suppresses HCC development when delivered using AAV vectors. In addition, miR-26a replacement induces cell-cycle arrest by inhibiting cyclins D2 and E2 in HCC cells [145]. Several potential targets, including miR-26a [146], miR-122 [352], and miR-124 [162], and promising strategies have been reported for miRNA replacement therapies. The inhibition of miR-221 results in a longer survival, reduction in nodule numbers, and retardation of tumor development [251,353]. Moreover, no toxicity was observed when miRNAs were used for the treatment of HCC in a mouse model. Interestingly, miRNAs influence the sensitivity of various cancers to anticancer drugs. Several studies have demonstrated that the patterns of miRNA expression are significantly changed after metformin treatment, in various cancers, such as gastric cancer [354], esophageal cancer [355], and HCC [356]. Notably, the enhanced expression of miR-21 [357] and miR-181b [242] induces chemoresistance to IFN–5-fluorouracil and doxorubicin therapy in HCC. By contrast, restoration of miR-122 as a tumor suppressor confers sensitivity to sorafenib to HCC cell lines via the downregulation of multidrug resistance genes.

### 4.5. miRNAs as Prognostic Markers for HCC

As shown in Table 2, recent studies have demonstrated that miRNAs, including miR-92a, miR-221, miR-487a, and miR-1468, have the potential to serve as prognostic biomarkers for HCC, and upregulation of these miRNAs indicates a poor prognosis for patients with HCC [112,125,130,203]. However, downregulation of miRNAs such as miR-33a, miR-137, miR-194, or miR-940 is also associated with a poor prognosis in HCC [142,148,172]. In addition, overexpression of exosomal miR-32-5p, which inhibits PTEN [204], and a low expression level of exosomal miR-638 [205] were found to be associated with a poor prognosis in patients with HCC. Furthermore, the expression levels of miR-122, miR-148a, and miR-1246 were found to be significantly higher in serum exosomes from patients with HCC than in those from patients with liver cirrhosis and from healthy controls [206].

## 5. Conclusions

Our understanding of biological functions of miRNAs and their diagnostic and therapeutic value in HCC is rapidly increasing. Various miRNAs have been identified to be involved in the modulation of biological processes during HCC development. In this review, we provided an overview of the roles of miRNAs in the development and progression of HCC. The promising findings for miRNA applications as HCC biomarkers and therapeutic targets indicate that (i) circulating miRNAs are stable in serum exosomes of patients with HCC and (ii) miRNAs can regulate multiple target genes and signaling pathways in HCC. Therefore, a combination of conventional molecular targeting agents and miRNA-based therapies for HCC might improve the efficiency of transgene expression and gene transfer in primary and metastatic HCC. Although miRNAs are potential biological target molecules for HCC diagnosis and therapy, the number of miRNA-based clinical trials is still insufficient. Major obstacles of the clinical application of miRNAs include inefficient drug delivery, off-target effects, and difficulties in the determination of efficacious doses. Site-specific delivery of miRNAs to HCC tissues and efficacious doses might minimize the off-target effects and toxicity. The precise targetable miRNAs and the mechanisms underlying the miRNA-induced anticancer effects in vivo remain unclear. However, innovative applications are increasing in various fields. Further analyses and new technologies for miRNAs will provide novel insights into the pathogenesis of HCC. Therefore, global miRNA analyses are expected to provide a basis for HCC therapy.

## Figures and Tables

**Table 1:** Downregulated and upregulated miRNAs in HCC.

miRNA	Target	Category	Expression	References
let-7a	Caspase-3, STAT3	apoptosis, proliferation	Down	[127,128,129]
let-7b	HMGA2	apoptosis, proliferation	Down	[130]
let-7c	Bcl-xL, c-myc	apoptosis, proliferation, cell growth	Down	[131,132,133]
let-7d		apoptosis, proliferation	Down	[128]
let-7f-1		apoptosis, proliferation	Down	[128]
let-7g	Bcl-xL, c-myc, COLIA2, p16	apoptosis, metastasis	Down	[134,135,136]
miR-1	ET-1	proliferation	Down	[137]
miR-7	PIK3CD		Down	[138]
miR-10a	EphA4		Down	[139]
miR-10b			Down	[140]
miR-15a/16			Down	[141]
miR-15b	BCL-2	proliferation, apoptosis	Down	[142]
miR-21			Down	[143]
miR-26a	CDK6, IL-6, cyclin D2, E1, E2	Cell cycle	Down	[144,145,146]
miR-29a	SPARC	proliferation	Down	[147]
miR-29b	MMP-2	invasion	Down	[148]
miR-29c	SIRT1		Down	[149]
miR-31-5p	SP1	proliferation, migration, invasion	Down	[150]
miR-34a	c-Met, CCL22	metastasis	Down	[71,151]
miR-98	EZH2	proliferation	Down	[152]
miR-99a	PLK1, IGF-1R		Down	[153,154]
miR-100	PLK1	carcinogenesis	Down	[153]
miR-101	EZH2, EED, Mcl-1, Fos	apoptosis, DNA methylation	Down	[155,156,157]
miR-122	Bcl-w, ADAM17, Wnt1	apoptosis, proliferation, angiogenesis	Down	[158,159,160,161]
miR-124	PIK3CA	proliferation	Down	[162]
miR-125a	MMP11, VEGF-A,SIRT7	proliferation, metastasis, metabolism	Down	[163,164]
miR-125b	Mcl-1, Bcl-w, SUV39H1, SIRT7	proliferation, metastasis, angiogenesis, apoptosis, histone modification	Down	[131,164,165,166,167,168]
miR-126	VEGF	angiogenesis	Down	[169]
miR-139	ROCK2, c-Fos	metastasis	Down	[131,170,171]
miR-139-5p			Down	[131]
miR-140-5p	TGFβR1, FIF9, DMMT1		Down	[172,173]
miR-141	DLC-1		Down	[95]
miR-142	TGFβ, THBS4	cell growth, metastasis, migration, invasion	Down	[174,175]
miR-142-3p	LDHA	proliferation	Down	[176]
miR-144	ZFX	proliferation, invasion, migration	Down	[177]
miR-145	IRS1/2, IGF-1R, β-catenin		Down	[178,179]
miR-148a	c-Met, HRIP, E-cadherin, c-Myc		Down	[180,181,182,183]
miR-152	DNMT1, GSTP1, CDH1		Down	[184]
miR-187-3p	S100A4	metastasis, EMT	Down	[185]
miR-194	MAP4K4	proliferation	Down	[112]
miR-195	cyclin D1, CDK6, E2F3, LATS2	cell cycle, tumorigenesis, apoptosis	Down	[186,187]
miR-198			Down	[39]
miR-199a-3p	mTOR, PAK4, caveolin-2	drug resistance, cell growth	Down	[188,189,190]
miR-199a-5p	DDR1, ATG7	invasion, autophagy	Down	[191,192]
miR-199b		proliferation, invasion, apoptosis	Down	[193]
miR-200a	HDAC4	proliferation, metastasis	Down	[194]
miR-200b			Down	[131]
miR-200c			Down	[143]
miR-203	Surviving	proliferation	Down	[195]
miR-206	Cyclin D1, CDK6	proliferation	Down	[196]
miR-212	FOXM1	migration, cell growth	Down	[197]
miR-214	HDGF, β-catenin	cell growth, angiogenesis, metastasis	Down	[198,199,200]
miR-219-5p	GPC3	proliferation	Down	[201]
miR-222			Down	[143]
miR-223	STMN1	proliferation	Down	[38]
miR-224			Down	[202]
miR-296	FGFR1	proliferation, apoptosis	Down	[203]
miR-302b	EGFR, AKT2	proliferation, invasion, metastasis	Down	[204]
miR-337	HMGA3	proliferation, apoptosis	Down	[205]
miR-338-3p	MACC1, β-catenin, VEGF	angiogenesis	Down	[206]
miR-340	JAK1	proliferation, invasion	Down	[207]
miR-345	IRF1	metastasis, EMT	Down	[208]
miR-363-3p	c-Myc		Down	[183]
miR-370	PIM1	cell growth, invasion	Down	[209]
miR-375	ATG7, AEG-1	autophagy	Down	[210,211]
miR-376a	PIK3R1	apoptosis, proliferation	Down	[212]
miR-429	Rab18		Down	[213]
miR-449	c-Met	proliferation, apoptosis	Down	[214]
miR-450a	DNMT3a	proliferation	Down	[215]
miR-451	IL6R	angiogenesis	Down	[216]
miR-497	RICTOR	proliferation, migration, invasion	Down	[217]
miR-520b/e	NIK, MEKK2, cyclin D1	cell growth, proliferation	Down	[218,219]
miR-539	FSCN1	migration, invasion	Down	[220]
miR-612	AKT2		Down	[221]
miR-637	STAT activation		Down	[222]
miR-638	SOX2, VEGF	invasion, EMT, angiogenesis	Down	[223]
miR-663a	HMGA2	proliferation, invasion	Down	[224]
miR-874	DOR	proliferation, metastasis	Down	[196]
miR-940	CXCR2	migration, invasion	Down	[225]
miR-1271	GLP3		Down	[140]
miR-1299	CDK6	proliferation	Down	[226]
miR-10a	EphA4; CADM1	EMT, metastasis	Up	[139,227]
miR-10b	CSMD 1	division, migration, invasion	Up	[228]
miR-17-5p	p38 pathway	migration	Up	[229]
miR-18a	ER1a	proliferation	Up	[230]
miR-21	PTEN; RHOB; PDCD4	metastasis, drug resistance	Up	[35,73]
miR-22	Era, IL-1a	carcinogenesis	Up	[231]
miR-23a	PGC-1a, G6PC	gluconeogenesis	Up	[50]
miR-25	TRAIL	apoptosis	Up	[119]
miR-26a	IL-6, CyclinD2, E2	tumor growth, metastasis	Up	[144,145]
miR-30d	GNAI2	invasion, metastasis	Up	[232]
miR-92a	FBXW 7	Proliferation, cell cycle transition, apoptosis	Up	[124]
miR-96-5p	Caspase-9	apoptosis	Up	[120]
miR-100	PLK1	carcinogenesis	Up	[39]
miR-106b	APC	proliferation	Up	[233]
miR-107	HMGCS 2	cell growth, EMT	Up	[122,234]
miR-130b	TP53INP1	cell growth, self-renewal	Up	[235]
miR-135a	FOXM1, MTSS1	metastasis	Up	[236]
miR-143	FNDC3B	metastasis	Up	[237]
miR-151	FAK, RhoGDIA	migration	Up	[238,239]
miR-155	SOCS1, DKK1, APC, PTEN	proliferation; Tumorigenesis	Up	[73,240,241]
miR-181b	TIMP3	tumorigenesis, metastasis	Up	[242]
miR-182	MTSS1	metastasis	Up	[243]
miR-183	AKAP12	carcinogenesis	Up	[244]
miR-186	AKAP12	carcinogenesis	Up	[244]
miR-200	NRF2 pathway	carcinogenesis	Up	[245]
miR-203a-3p	Interleukin (IL) 24	cell growth, metastasis	Up	[246]
miR-210	VMP1	metastasis	Up	[247]
miR-214-5p	WASL	Invasion, migration	Up	[248]
miR-216a	TSLC1	carcinogenesis	Up	[249]
miR-216a/217	PTEN, SMAD7	EMT, drug resistance	Up	[250]
miR-221	p27, p57, Arnt, CDK inhibitors	apoptosis, proliferation, angiogenesis	Up	[251,252,253]
miR-221/222	p27, DDIT4	tumorigenesis	Up	[254]
miR-224	Atg5, Smad4, autophagy, API-5	tumorigenesis, autophagy	Up	[255,256]
miR-301a	Gax	metastasis	Up	[257]
miR-302d	TGFβRII	cell growth, invasion,	Up	[258]
miR-346	FBXL2	proliferation, migration, invasion	Up	[259]
miR-373	PPP6C	cell cycle	Up	[260]
miR-423	p21/waf1	cell growth	Up	[261]
miR-485-3p	MAT1, LIN28B	cell growth, EMT	Up	[262]
miR-487a	SPRED2, PIK3R1	proliferation, metastasis	Up	[263]
miR-490-3p	ERCIC3	EMT	Up	[264]
miR-494	MCC	tumorigenesis	Up	[265]
miR-495	MAT1, LIN28B	tumorigenesis, metastasis	Up	[262]
miR-517a		tumorigenesis, metastasis	Up	[266]
miR-519d	p21, PTEN, AKT3, TIMP2	proliferation, invasion, apoptosis	Up	[267]
miR-550a	CPEB4	proliferation, invasion, metastasis	Up	[268]
miR-590-5p	TGFβRII	metastasis, proliferation	Up	[269]
miR-615-5p	IGF-II	proliferation, migration	Up	[270]
miR-657	TLE1, NF–κB	proliferation	Up	[271]
miR-664	MAT1, LIN28B	tumorigenesis, metastasis	Up	[262]
miR-765			Up	[272]
miR-873			Up	[273]
miR-892a			Up	[274]
miR-1249			Up	[275]
miR-1323		proliferation	Up	[276]
miR-3910			Up	[277]
miR-4417			Up	[278]

**Table 2:** miRNAs as biomarkers for HCC.

	miRNA	Expression	Etiology	Reference
Diagnosis	let-7g	down	HBV	[135]
	miR-21	up	HBV, HCV and others	[15,287]
	miR-22	down	HBV	[331]
	miR-99a	down	HBV, HCV and others	[154]
	miR-101	down	HBV	[332]
	miR-122	down	HBV, HCV	[333,344]
	miR-124	down	HBV, HCV and others	[334]
	miR-125a-5p	up	HBV, HCV and others	[345]
	miR-135a	up	HBV, HCV and others	[236]
	miR-139	down	HBV and others	[335]
	miR-155	up	HBV	[341]
	miR-182	up	HBV and others	[243]
	miR-199b	down	HBV, HCV and others	[193]
	miR-221	up	HBV, HCV and others	[340]
	miR-222	up	HBV, HCV and others	[170,340]
	miR-223-3p	down	HBV	[345]
	miR-375	up	HBV	[335]
Poor prognosis	miR-92a	up	HBV and others	[124]
	miR-122	up	HBV, HCV	[234]
	miR-148a	up	HBV, HCV	[234]
	miR-221	up	HBV and others	[346]
	miR-296	down	HBV and others	[203]
	miR-487a	up	HBV and others	[263]
	miR-638	down	HBV, HCV and others	[347]
	miR-940	down	HBV and others	[225]
	miR-1246	up	HBV, HCV	[234]
