# Correction: Morishita et al. MicroRNAs in the Pathogenesis of Hepatocellular Carcinoma: A Review. *Cancers* 2021, *13*, 514

**DOI:** 10.3390/cancers18020232

**Published:** 2026-01-12

**Authors:** Asahiro Morishita, Kyoko Oura, Tomoko Tadokoro, Koji Fujita, Joji Tani, Tsutomu Masaki

**Affiliations:** Department of Gastroenterology and Neurology, Faculty of Medicine, Kagawa University, Kagawa 761-0793, Japan; kyoko_oura@med.kagawa-u.ac.jp (K.O.); t-nishioka@med.kagawa-u.ac.jp (T.T.); 92m7v9@med.kagawa-u.ac.jp (K.F.); georget@med.kagawa-u.ac.jp (J.T.); tmasaki@med.kagawa-u.ac.jp (T.M.)

Following publication [1], concerns were raised to the Editorial Office relating to various citation concerns. Adhering to our standard policy, an investigation was completed by the Editorial Office and Editorial Board which confirmed that a number of the citations have been retracted following the publication of this paper.

After discussion with the authors, the Editorial Board have decided that a correction to remove and include replacement/additional citations could appropriately address this matter and ensure the overall validity of the findings of this study. Therefore, with the publication of this correction, 18 citations originally published have been removed, 13 new citations have been added and the citation numbers have been adjusted accordingly. Details related to the removed and replacement citations can be found below. The previously mismatched order of the references has also been updated. The authors state that the scientific conclusions are unaffected. This correction was approved by the Academic Editor. The original publication has also been updated.

## Additional Citations with Updated Citation Numbers

79. Jinato, T.; Chuaypen, N.; Poomipak, W.; Praianantathavorn, K.; Makkoch, J.; Kiatbumrung, R.; Jampoka, K.; Tangkijvanich, P.; Payungporn, S. Original Research: Analysis of hepatic microRNA alterations in response to hepatitis B virus infection and pegylated interferon alpha-2a treatment. *Exp. Biol. Med.* **2016**, *241*, 1803–1810.
80. Zhao, X.; Sun, L.; Mu, T.; Yi, J.; Ma, C.; Xie, H.; Liu, M.; Tang, H. An HBV-encoded miRNA activates innate immunity to restrict HBV replication. *J. Mol. Cell Biol.* **2020**, *12*, 263–276.
83. Wang, J.; Liu, X.; Wu, H.; Ni, P.; Gu, Z.; Qiao, Y.; Chen, N.; Sun, F.; Fan, Q. CREB up-regulates long non-coding RNA, HULC expression through interaction with microRNA-372 in liver cancer. *Nucleic Acids Res.* **2010**, *38*, 5366–5383.
100. Bhanja Chowdhury, J.; Shrivastava, S.; Steele, R.; Di Bisceglie, A.M.; Ray, R.; Ray, R. B. Hepatitis C virus infection modulates expression of interferon stimulatory gene IFITM1 by upregulating miR-130A. *J. Virol.* **2012**, *86*, 10221–10225.
114. Khanizadeh, S.; Ravanshad, M.; Hosseini, S.Y.; Davoodian, P.; Zadeh, A.N.; Sabahi, F.; Sarvari, J.; Khanlari, Z.; Hasani-Azad, M. The possible role of NS3 protease activity of hepatitis C virus on fibrogenesis and miR-122 expression in hepatic stellate cells. *Acta Virol.* **2016**, *60*, 242–248.
281. Niu, X.; Sun, H.; Qiu, F.; Liu, J.; Yang, T.; Han, W. miR-10b-5p Suppresses the Proliferation and Invasion of Primary Hepatic Carcinoma Cells by Downregulating EphA2. *Biomed. Res. Int.* **2021**, *2021*, 1382061.
282. Dou, C.; Liu, Z.; Xu, M.; Jia, Y.; Wang, Y.; Li, Q.; Yang, W.; Zheng, X.; Tu, K.; Liu, Q., miR-187-3p inhibits the metastasis and epithelial-mesenchymal transition of hepatocellular carcinoma by targeting S100A4. *Cancer Lett.* **2016**, *381*, 380–390.
283. Zhou, S.J.; Liu, F.Y.; Zhang, A.H.; Liang, H.F.; Wang, Y.; Ma, R.; Jiang, Y.H.; Sun, N.F. MicroRNA-199b-5p attenuates TGF-beta1-induced epithelial-mesenchymal transition in hepatocellular carcinoma. *Br. J. Cancer* **2017**, *117*, 233–244.
284. Yang, Q.; Zhang, L.; Zhong, Y.; Lai, L.; Li, X. miR-206 inhibits cell proliferation, invasion, and migration by down-regulating PTP1B in hepatocellular carcinoma. *Biosci. Rep.* **2019**, *39*, BSR20181823.
285. Zhang, Y.; Takahashi, S.; Tasaka, A.; Yoshima, T.; Ochi, H.; Chayama, K. Involve ment of microRNA-224 in cell proliferation, migration, invasion, and anti-apoptosis in hepatocellular carcinoma. *J. Gastroenterol. Hepatol.* **2013**, *28*, 565–575.
286. Huo, W.; Du, M.; Pan, X.; Zhu, X.; Gao, Y.; Li, Z. miR-203a-3p.1 targets IL-24 to modulate hepatocellular carcinoma cell growth and metastasis. *FEBS Open. Bio.* **2017**, *7*, 1085–1091.
313. Azar, F.; Courtet, K.; Dekky, B.; Bonnier, D.; Dameron, O.; Colige, A.; Legagneux, V.; Theret, N. Integration of miRNA-regulatory networks in hepatic stellate cells identifies TIMP3 as a key factor in chronic liver disease. *Liver Int.* **2020**, *40*, 2021–2033.
327. Zhang, P.; Yin, J.; Yuan, L.; Wang, Q.; Du, X.; Dong, R.; Wang, C.; Bai, Q.; Ji, L.; Zhang, G.; et al. MicroRNA-139 suppresses hepatocellular carcinoma cell proliferation and migration by directly targeting Topoisomerase I. *Oncol. Lett.* **2019,** *17*, 1903–1913.

## Removed Citations

80. Fisicaro, P.; Valdatta, C.; Boni, C.; Massari, M.; Mori, C.; Zerbini, A.; Orlandini, A.; Sacchelli, L.; Missale, G.; Ferrari, C. Early kinetics of innate and adaptive immune responses during hepatitis B virus infection. *Gut* **2009**, *58*, 974–982. https://doi.org/10.1136/gut.2008.163600.
81. Mizuguchi, Y.; Takizawa, T.; Yoshida, H.; Uchida, E. Dysregulated miRNA in progression of hepatocellular carcinoma: A systematic review. *Hepatol. Res.* **2016**, *46*, 391–406. https://doi.org/10.1111/hepr.12606.
82. Bertoletti, A.; Kennedy, P.T.F. The immune tolerant phase of chronic HBV infection: New perspectives on an old concept. *Cell. Mol. Immunol.* **2014**, *12*, 258–263. https://doi.org/10.1038/cmi.2014.79.
100. Chowdhury, J.B.; Shrivastava, S.; Steele, R.; Di Bisceglie, A.M.; Ray, R.; Ray, R.B. Hepatitis C Virus Infection Modulates Expression of Interferon Stimulatory Gene IFITM1 by Upregulating miR-130A. *J. Virol.* **2012**, *86*, 10221–10225. https://doi.org/10.1128/jvi.00882-12.
109. Wan, Y.; McDaniel, K.; Wu, N.; Ramos-Lorenzo, S.; Glaser, T.; Venter, J.; Francis, H.; Kennedy, L.; Sato, K.; Zhou, T.; et al. Regulation of Cellular Senescence by miR-34a in Alcoholic Liver Injury. *Am. J. Pathol.* **2017**, *187*, 2788–2798. https://doi.org/10.1016/j.ajpath.2017.08.027.
115. Wu, F.-L.; Jin, W.-B.; Li, J.-H.; Guo, A.-G. Targets for human encoded microRNAs in HBV genes. *Virus Genes* **2011**, *42*, 157–161. https://doi.org/10.1007/s11262-010-0555-7.
152. Tian, Q.; Xiao, Y.; Wu, Y.; Liu, Y.; Song, Z.; Gao, W.; Zhang, J.; Yang, J.; Zhang, Y.; Guo, T.; et al. MicroRNA-33b suppresses the proliferation and metastasis of hepatocellular carcinoma cells through the inhibition of Sal-like protein 4 expression. *Int. J. Mol. Med.* **2016**, *38*, 1587–1595. https://doi.org/10.3892/ijmm.2016.2754. Retracted in *Int. J. Mol. Med.* **2021**, *48*, 170.
172. Cui, S.; Sun, Y.; Liu, Y.; Liu, C.; Wang, J.; Hao, G.; Sun, Q. MicroRNA-137 has a suppressive role in liver cancer via targeting EZH2. *Mol. Med. Rep.* **2017**, *16*, 9494–9502. https://doi.org/10.3892/mmr.2017.7828. Retracted in *Mol. Med. Rep.* **2022**, *25*, 145.
208. Wang, L.; Yao, J.; Sun, H.; Sun, R.; Chang, S.; Yang, Y.; Song, T.; Huang, C. miR-302b suppresses cell invasion and metastasis by directly targeting AKT2 in human hepatocellular carcinoma cells. *Tumor Biol.* **2016**, *37*, 847–855.
221. Ye, Y.; Zhuang, J.; Wang, G.; He, S.; Zhang, S.; Wang, G.; Ni, J.; Wang, J.; Xia, W. MicroRNA 495 suppresses cell proliferation and invasion of hepatocellular carcinoma by directly targeting insulin like growth factor receptor 1. *Exp. Ther. Med.* **2018**, *15*, 1150–1158. https://doi.org/10.3892/etm.2017.5467. Retracted in *Exp Ther Med.* **2022**, *24*, 505.
232. Yang, C.; Xu, Y.; Cheng, F.; Hu, Y.; Yang, S.; Rao, J.; Wang, X. miR-1301 inhibits hepatocellular carcinoma cell migration, invasion, and angiogenesis by decreasing Wnt/β-catenin signaling through targeting BCL9. *Cell Death Dis.* **2017**, *8*, e2999. https://doi.org/10.1038/cddis.2017.356. Retracted in *Cell Death Dis.* **2023**, *14*, 225.
283. Liu, Z.; Wang, Y.; Dou, C.; Sun, L.; Li, Q.; Wang, L.; Xu, Q.; Yang, W.; Liu, Q.; Tu, K. MicroRNA-1468 promotes tumor progression by activating PPAR-γ-mediated AKT signaling in human hepatocellular carcinoma. *J. Exp. Clin. Cancer Res.* **2018**, *37*, 49. https://doi.org/10.1186/s13046-018-0717-3. Retracted in *J. Exp. Clin. Cancer Res.* **2022**, *41*, 272.
295. Yoon, J.S.; Kim, G.; Lee, Y.R.; Park, S.Y.; Tak, W.Y.; Kweon, Y.O.; Park, J.G.; Lee, H.W.; Han, Y.S.; Ha, H.T.; et al. Clinical significance of microRNA-21 expression in disease progression of patients with hepatocellular carcinoma. *Biomark. Med.* **2018**, *12*, 1105–1114. https://doi.org/10.2217/bmm-2018-0096.
309. Garofalo, M.; Di Leva, G.; Romano, G.; Nuovo, G.; Suh, S.-S.; Ngankeu, A.; Taccioli, C.; Pichiorri, F.; Alder, H.; Secchiero, P.; et al. miR-221&222 Regulate TRAIL Resistance and Enhance Tumorigenicity through PTEN and TIMP3 Downregulation. *Cancer Cell* **2009**, *16*, 498–509. https://doi.org/10.1016/j.ccr.2009.10.014. Retracted in *Cancer Cell* **2022**, *40*, 1440.
315. Yuan, B.; Dong, R.; Shi, D.; Zhou, Y.; Zhao, Y.; Miao, M.; Jiao, B. Down-regulation of miR-23b may contribute to activation of the TGF-β1/Smad3 signalling pathway during the termination stage of liver regeneration. *FEBS Lett.* **2011**, *585*, 927–934. https://doi.org/10.1016/j.febslet.2011.02.031.
340. Guo, Y.; Xiong, Y.; Sheng, Q.; Zhao, S.; Wattacheril, J.; Flynn, C.R. A micro-RNA expression signature for human NAFLD progression. *J. Gastroenterol.* **2016**, *51*, 1022–1030.
345. Li, L.-M.; Hu, Z.-B.; Zhou, Z.-X.; Chen, X.; Liu, F.-Y.; Zhang, J.-F.; Shen, H.-B.; Zhang, C.-Y.; Zen, K. Serum microRNA Profiles Serve as Novel Biomarkers for HBV Infection and Diagnosis of HBV-Positive Hepatocarcinoma. *Cancer Res.* **2010**, *70*, 9798–9807. https://doi.org/10.1158/0008-5472.can-10-1001.
350. Fu, X.; Liu, M.; Qu, S.; Ma, J.; Zhang, Y.; Shi, T.; Wen, H.; Yang, Y.; Wang, S.; Wang, J.; et al. Exosomal microRNA-32-5p induces multidrug resistance in hepatocellular carcinoma via the PI3K/Akt pathway. *J. Exp. Clin. Cancer Res. ***2018**, *37*, 52. https://doi.org/10.1186/s13046-018-0677-7. Retracted in *J. Exp. Clin. Cancer Res.* **2023**, *42*, 174.
